# Grape Seed Proanthocyanidin Rescues Rats from Steatosis: A Comparative and Combination Study with Metformin

**DOI:** 10.1155/2013/153897

**Published:** 2013-11-06

**Authors:** Baskaran Yogalakshmi, S Sreeja, Rajagopalan Geetha, Mutlur Krishnamoorthy Radika, Carani Venkatraman Anuradha

**Affiliations:** Department of Biochemistry and Biotechnology, Annamalai University, Annamalai Nagar, Tamil Nadu 608 002, India

## Abstract

Nonalcoholic fatty liver disease (NAFLD), a premorbid condition, lacks proper management owing to multitude of abnormalities. In this study, we compared the effects of a potent antioxidant, grape seed proanthocyanidins (GSP), and an insulin sensitizer, metformin (MET), in high-fat-fructose-diet- (HFFD-) induced albino Wistar rat model of NAFLD. Either GSP (100 mg/Kg b.w) or MET (50 mg/Kg b.w) or both were administered as therapeutic options. HFFD-fed rats showed abnormal plasma lipid profile, inflammation, and steatosis of the liver when examined by biochemical and histology techniques. Increased lipid storage, lipogenesis, and reduced lipolysis were evident from mRNA expression studies of hepatic lipid droplets (LD) proteins, sterol regulatory element binding 1c (SREBP 1c), and peroxisome proliferator activated receptor-**α** (PPAR-**α**). GSP administration to HFFD-fed rats caused 69% reduction in hepatic TG levels, whereas MET caused only 23%. The combination treatment reduced TG levels by 63%. GSP reduced the mRNA expression of SREBP1c and LD proteins and increased that of PPAR-**α** more effectively compared to MET in HFFD-induced hyperlipidemic rats. Combination of MET and GSP improved the metabolism of lipids effectively, but the effect was not additive in restoring lipid levels.

## 1. Introduction

Nonalcoholic fatty liver disease (NAFLD) has become a global health problem in both adults and children, the prevalence of which has doubled during last 20 years in tune with the increasing incidence of obesity and insulin resistance [1]. Improper regulation of lipogenesis and lipid oxidation is a prime factor in the genesis of fatty liver. 

Lipogenesis encompasses the processes of fatty acid synthesis and subsequent formation of triglyceride (TG). Sterol regulatory elemental binding protein-1c (SREBP-1c) belongs to a family of transcription factors that are sensitive to cellular availability of cholesterol [2] and promotes fatty acid synthesis and lipid deposition. SREBP-1c regulates lipogenesis by increasing the expression of genes involved in fatty acid biosynthesis such as acetyl CoA carboxylase, fatty acid synthase, and stearoyl CoA desaturase. SREBP-1c has been implicated in the development of human metabolic disorders like obesity, type 2 diabetes, dyslipidemia, atherosclerosis, lipodystrophy, and metabolic syndrome.

Lipid reserves are stored in the form of lipid droplets (LDs) intracellularly. LDs, long considered as inert substances, are now recognized for their dynamic role in lipid metabolism. They are structurally similar to circulating lipoproteins, consisting of a core of esterified lipids (TG, cholesterol ester, retinol esters, or ether lipids, depending on the cell type) covered by a phospholipid monolayer, free cholesterol, and a coat of specific proteins [3, 4]. The LD proteins include the founding member perilipin (plin1), adipophilin (plin2, also known as adipocyte differentiation-related protein (ADRP)), tail-interacting protein of 47 kilo daltons (TIP47, plin3), S3-12 (plin4), and myocardial lipid droplet protein (plin5) [5]. These are collectively known as the PAT family of proteins named after perilipin, adipophilin, and TIP47. Another protein termed fat-specific protein 27 (FSP27), although not a member of PAT family proteins, is recognized to be a structural protein present in LD and is specifically involved in diet-induced fatty liver [6]. LD proteins are involved in lipid metabolism and transport, intracellular trafficking, signaling, and cytoskeletal organization [7–9]. LD proteins also regulate lipolysis in adipose tissue by modulating the access of hormone sensitive lipase (HSL) to lipid surface [7]. The LD proteins also serve to regulate lipolysis of TG rich droplets in response to insulin and adrenergic stimulation [10]. LD proteins are suggested to play a role in the pathophysiology of NAFLD [11].

Peroxisome proliferator-activated receptor-*α* (PPAR-*α*) is a nuclear receptor protein and is the first member of PPAR family cloned in 1990 [12]. Among the three isoforms of PPAR family-*α*, *β*, and *γ*, PPAR-*α* is predominantly present in liver and is involved in the activation of hepatic lipid catabolism by targeting genes involved in cellular fatty acid uptake [13] and transport [14], mitochondrial and peroxisomal fatty acid uptake, and *β*-oxidation [15]. Among the various endogenous ligands for PPAR-*α*, long-chain fatty acids are the most studied. Thus PPAR-*α* serves as a lipid sensor and directs lipid metabolism towards oxidation when upregulated by agonists.

Grape seed proanthocyanidins (GSP) is a complex mixture of oligomeric compounds possessing high antioxidant activity with preventive effects on some forms of cancer and oxidative injury [16]. Proanthocyanidin is richly present in red wine, and its content in red wine is reported to be 117.18 ± 96.06 mg/L [17]. The first clue to its lipid-lowering effect came from the French paradox, which refers to the lower mortality rates of cardiovascular disease despite high intake of saturated fats due to red wine consumption. In fact studies have shown that GSP can lower postprandial plasma TGs and apolipoproteins levels in healthy rats [18]. 

The insulin-sensitizing agent metformin (MET) is used as a treatment strategy in NAFLD patients due to the role of insulin resistance in the pathogenesis of NAFLD. Since NAFLD is a multifactorial disease, combination therapy is recommended to address the individual components like hyperlipidemia and insulin resistance. We hypothesize that GSP may help to increase the oxidation of lipids by reducing the lipogenic pathway and lipid storage while MET may improve the hepatic insulin sensitivity. Hence, in this study, we compared the effects of GSP and MET individually and in combination against diet-induced NAFLD.

## 2. Materials and Methods

### 2.1. Experimental Diet and Treatment

Proanthocyanidin-rich extract from grape seed (GSP, gravinol-Super^TM^) was kindly provided by Kikkoman Co. (Chiba, Japan). The grape seed extract contained 89% proanthocyanidin, 6% monomers, and 5% other materials. Metformin hydrochloride was obtained from the local drug store (Ranbaxy, India). The normal rat feed was obtained from Sai enterprise, Chennai, India, which contained 60% starch, 22.08% protein, and 4.38% fat. This commercial diet provided 382.61 cal/100 g. The high-fat/fructose diet (HFFD) prepared in our laboratory contained 45% fructose, 20% fat, (10% beef tallow, 10% groundnut oil), and 22.5% casein and provided 471.25 cal/100 g.

### 2.2. Experimental Animals and Study Design

This study was conducted in strict accordance with the guidelines of the Committee for the Purpose of Control and Supervision on Experiments on Animals (CPCSEA). All procedures were approved and adhered to the guidelines of the Institutional Animal Ethical Committee (IAEC). Six-week-old male albino Wistar rats (*n* = 6) were purchased from Rajah Muthiah Medical College and Hospital (RMMC and H, Annamalai University) and left for acclimatization in the department animal house for a week. Obesity was induced in rats by feeding HFFD. Therapeutic intervention with GSP or MET or combination of both was initiated after 30 days of HFFD feeding. GSP (100 mg/kg b.w/day in water) and MET (50 mg/kg b.w/day in water) were administered orally once a day to the respective groups from the 31st day till the 45th day. For combination treatment, GSP and MET were administered at an interval of 4 hours. At the end of the experiment, the rats were fasted for 18 h. After decapitation, blood samples were collected in tubes containing EDTA and centrifuged to obtain plasma. Liver was removed immediately and washed with ice cold physiological saline. Samples were stored at −80°C until further analysis. 

### 2.3. Biochemical Analysis

Oral glucose tolerance test (OGTT) was carried out on the 44th day after an overnight fast (12 hours) as described elsewhere [19]. For this, animals were given glucose (2 g/kg b.w, oral) after collecting fasting blood samples. Additional blood samples were drawn after one hour and two hours by sinoocular puncture in heparinized test tubes and centrifuged at 3000 ×g for 10 minutes to separate plasma. Glucose was measured using a kit (Agappe Diagnostics Pvt, Ltd., Kerala, India). Plasma insulin was assayed using an enzyme-linked immunosorbent assay kit (Accubind, Monobind Chemicals, Ltd., Lake Forest, CA). Insulin sensitivity was assessed by computing insulin sensitivity index (ISI_0,120_) [20]. 

Lipids in plasma and tissues were extracted by the method of Folch and colleagues [21]. Total lipids, extracted with chloroform-methanol mixture (2 : 1 (v/v)) from liver, were evaporated to dryness and used for the estimation. Estimation of cholesterol, TG, and FFAs in plasma and liver was carried out following procedures described earlier [22]. High density lipoprotein cholesterol (HDL-C) was determined following the kit procedure (Agappe diagnostics) and low density lipoprotein cholesterol (LDL-C) and very low density lipoprotein cholesterol (VLDL-C) were calculated using the following equations: VLDL (mg/dL) = TG/5; LDL (mg/dL) = Total Cholesterol−(HDL-C + VLDL-C) [23]. All analyses were completed within 24 h of sample collection.

### 2.4. Quantitative Polymerase Chain Reaction on LD Genes, SREBP-1c, HMG CoA Reductase, and PPAR-*α*

Total RNA was isolated from rat liver using the Trizol reagent (Invitrogen, Carlsbad, CA, USA). RNA concentration was assessed in a Biophotometer (Eppendorf, Hamburg, Germany) and reverse-transcribed using M-MuLV-reverse transcriptase (Fermentas) and Oligo dT primers (Invitrogen). cDNA was quantified using spectrophotometer (Eppendorf) and amplified using the Maxima SYBR Green qPCR master mix (Fermentas, Pittsburgh, USA). Amplification of target genes detailed in Table 1 was performed using realplex mastercycler (Eppendorf) using GAPDH as endogenous control. All quantifications were performed in triplicate samples for three separate experiments. The amount of target gene, normalized to GAPDH and relative to a calibrator, was determined by the arithmetic formula 2^−ΔΔCt^ by the comparative Ct method.

### 2.5. Histopathological Examinations

Histologic analyses of liver were performed after liver tissue samples were fixed at room temperature in 4% formaldehyde and embedded in paraffin. Five-micrometer sections were mounted on glass slides, deparaffinized in xylol, and stained for hematoxylin and eosin to evaluate steatosis and inflammation. Localization of lipids was performed by Oil Red O staining of frozen-liver sections, followed by counterstaining with hematoxylin for visualization of the nuclei.

### 2.6. Statistical Analysis

Values are presented as means ± SD. All data analysis was performed with the use of SPSS statistical software 17.0. The statistical significance of differences between groups was determined by one-way ANOVA followed by the Duncan's multiple comparison tests. *P* < 0.05 was considered to indicate a statistically significant result.

## 3. Results

**Figure 1:**
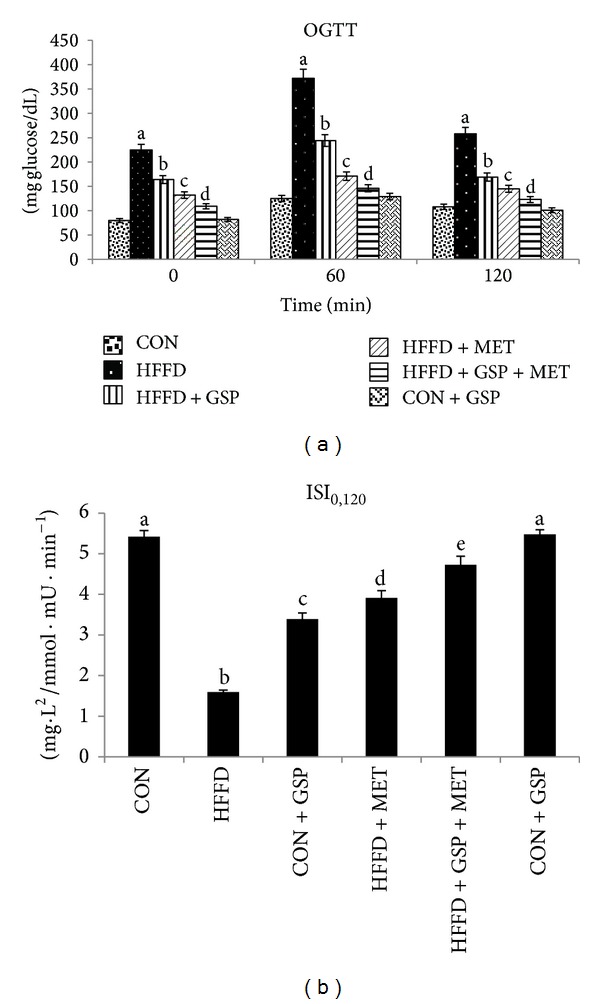
Effect of GSP and MET on oral glucose tolerance test (a) and insulin sensitivity indices (b). Data are expressed as means ± S.D. of 6 rats from each group. Statistical significance between the groups, denoted by different alphabets, was determined by one-way ANOVA of significance set at *P* < 0.05.

OGTT results (Figure 1(a)) show a rise in glucose levels in HFFD-fed rats by 2.8-, 2.9-, and 2.3-fold in plasma samples collected at 0, 60, and 120 min, respectively, after an oral glucose challenge compared to CON. All three treatments showed improved tolerance to glucose. Combination of GSP and MET showed a normal response and an additive improvement towards glucose load.

ISI_0,120_ (Figure 1(b)), a measure of insulin sensitivity, assessed using the OGTT values, was significantly decreased in HFFD-fed rats as compared to CON rats. Individual administration of GSP and MET improved insulin sensitivity. The values of ISI_0,120_ were 2.13- and 2.45-fold, respectively, in GSP and MET group compared to HFFD. Combination of GSP and MET showed additive improvement in ISI values (by 2.97-fold) compared to HFFD.

**Figure 2:**
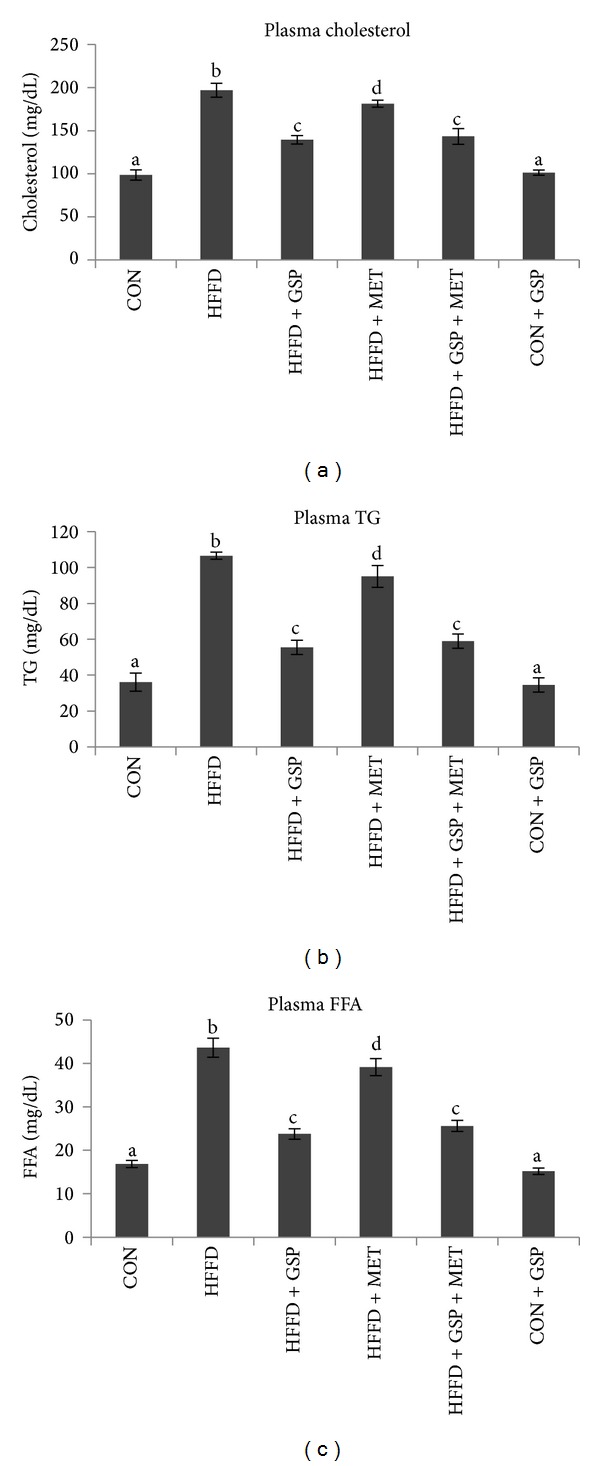
Effect of GSP and MET on levels of cholesterol (a), TG (b), and FFA (c) in plasma of experimental animals. Data are expressed as means ± S.D. of 6 rats from each group. Statistical significance between the groups, denoted by different alphabets, was determined by one-way ANOVA of significance set at *P* < 0.05.

**Figure 3:**
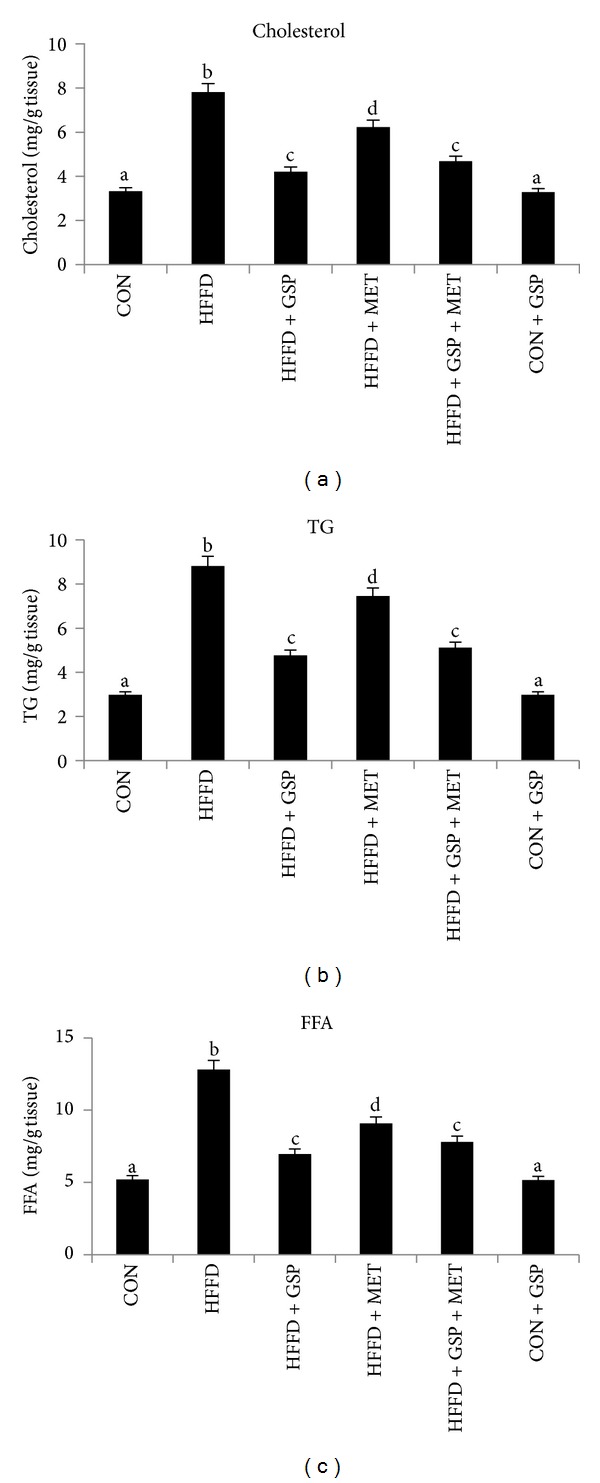
Effect of GSP and MET on levels of cholesterol (a), TG (b), and FFA (c) in liver of experimental animals. Data are expressed as means ± S.D. of 6 rats from each group. Statistical significance between the groups, denoted by different alphabets, was determined by one-way ANOVA of significance set at *P* < 0.05.

Plasma and hepatic cholesterol, TG, and FFA levels are given in Figures 2(a)–2(c) and Figures 3(a)–3(c), respectively. HFFD-fed rats showed increased cholesterol (2-fold in plasma and 2.3-fold in liver), TG (3-fold in plasma and liver), and FFA (2.6-fold in plasma and 2.5-fold in liver) compared to CON rats. These were reduced effectively and significantly after treatment with GSP in plasma (by 29%, 49%, and 45% of cholesterol, TG and FFA, resp.) and liver (by 46%, 45%, and 45% of cholesterol, TG and FFA, resp.) compared to HFFD-fed rats. MET was not much effective compared to GSP although it reduced the lipids levels in the plasma (by 8%, 10%, and 10% in cholesterol, TG and FFA, resp.) and liver (by 25%, 29%, and 15% of cholesterol, TG and FFA, resp.) compared to HFFD group. Combination of MET with GSP in HFFD-fed rats showed marked improvement in plasma (by 27%, 44% and 41% of cholesterol, TG and FFA, resp.) and liver (by 40%, 38%, and 42% of cholesterol, TG and FFA, resp.) compared to HFFD group of rats.

**Figure 4:**
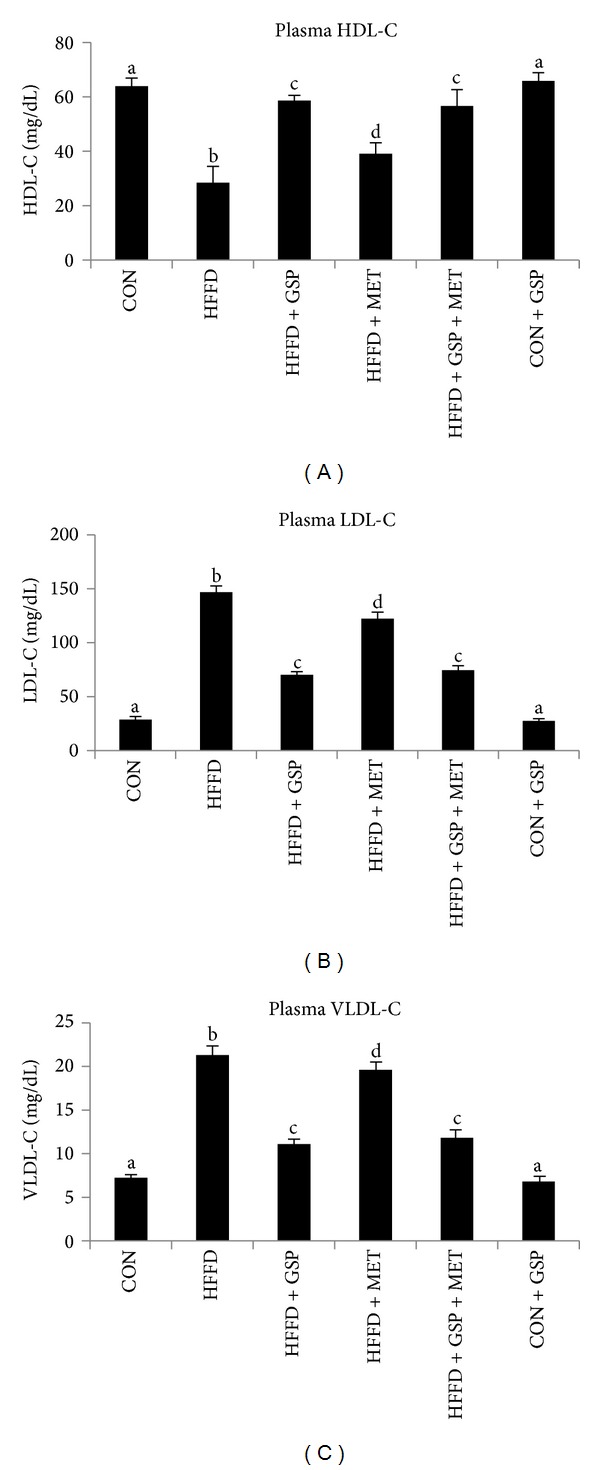
Effect of GSP and MET on levels of HDL-C (a), LDL-C (b), and VLDL-C (c) in liver of experimental animals. Data are expressed as means ± S.D. of 6 rats from each group. Statistical significance between the groups, denoted by different alphabets, was determined by one-way ANOVA of significance set at *P* < 0.05.

Plasma lipoproteins, namely, HDL-C, LDL-C, and VLDL-C were analyzed, and the values are presented in Figures 4(a)–4(c). HFFD-fed rats showed 55% reduction in HDL-C and 5.1- and 3-fold increase in LDL-C and VLDL-C values, respectively, compared to control rats. Compared to that of HFFD-fed rats, 0.9- fold increase in HDL-C and 52% and 47% decrease in the LDL-C and VLDL-C were observed during GSP treatment. MET showed 37% improvement in HDL-C and 16% and 8% decrease in LDL-C and VLDL-C, respectively, whereas the combination of MET with GSP showed similar effects to that of GSP alone compared to HFFD-fed rats (99% increase in HDL-C and 49% and 44% decrease in LDL-C, and VLDL-C resp.). 

**Figure 5:**
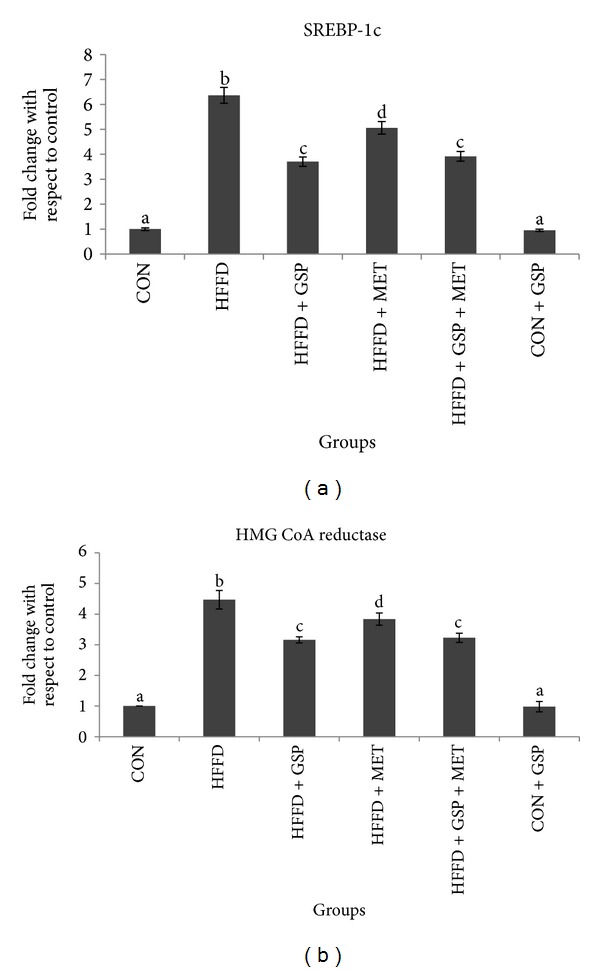
Effect of GSP and MET on mRNA expression of lipogenesis markers in liver of experimental animals. mRNA of SREBP-1c (a) and mRNA of HMG CoA reductase (b). Experiments were performed in triplicates, and the data expressed are means ± S.D. of 3 rats from each group. The obtained Ct values of the test genes were normalized with GAPDH and expressed in bars as fold change. Statistical significance between the groups, denoted by different alphabets, was determined by one-way ANOVA of significance set at *P* < 0.05.

mRNA expression of the lipogenic transcription factor, SREBP-1c (Figure 5(a)), and the rate limiting enzyme in cholesterol biosynthesis and HMG CoA reductase (Figure 5(b)), was found to be 6.4- and 4.5-fold, respectively, in liver of HFFD-fed rats compared to CON rats. This increase was attenuated significantly by GSP and MET supplementation. With respect to CON, the expression levels of SREBP1c and HMG CoA reductase were 3.7- and 3.2- fold in GSP and 5- and 3.8-fold in MET, respectively. Combination of MET and GSP showed similar effects as that of GSP in mRNA expression of SREBP-1c and HMG CoA reductase (see Figures 5A and 5B in Supplementary Material available online at http://dx.doi.org/10.1155/2013/153897).

**Figure 6:**
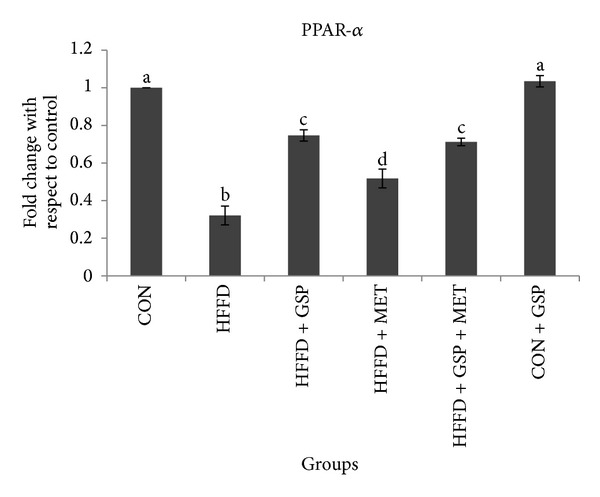
Effect of GSP and MET on mRNA expression of fatty acid oxidation marker PPAR-*α* in liver of experimental animals. Experiments were performed in triplicates, and the data expressed are means ± S.D. of 3 rats from each group. The obtained Ct values of the test gene were normalized with GAPDH and expressed in bars as fold change. Statistical significance between the groups, denoted by different alphabets, was determined by one-way ANOVA of significance set at *P* < 0.05.

mRNA expression of the fat sensor and PPAR-*α* was found to be markedly lower (by 68%) in liver of HFFD-fed rats compared to CON (Figure 6). This decrease was attenuated by GSP supplementation to HFFD rats. In HFFD + GSP, the expression was improved by 56% compared to HFFD indicating improved fatty acid oxidation despite continuous HFFD feeding. mRNA expression of PPAR-*α* in MET administered rats was improved by 37% compared to HFFD which is less than that induced by GSP alone. In MET and GSP treatment group, PPAR-*α* expression increased by 54% with respect to HFFD group (see Figure 6 in Supplementary Material available online at http://dx.doi.org/10.1155/2013/153897).

**Figure 7:**
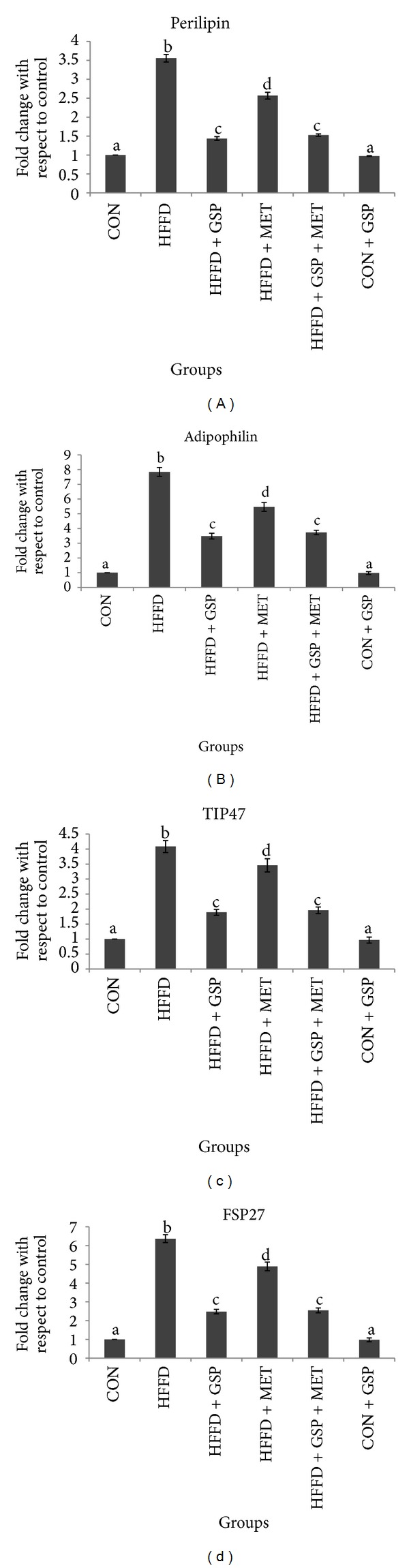
Effect of GSP and MET on mRNA expression of LD proteins perilipin (a), adipophilin (b), TIP47 (c), and FSP27 (d) in liver of experimental animals. Experiments were performed in triplicates and the data expressed are means ± S.D. of 3 rats from each group. The obtained Ct values of the test genes were normalized with GAPDH and expressed in bars as fold change. Statistical significance between the groups, denoted by different alphabets, was determined by one-way ANOVA of significance set at *P* < 0.05.

Increase in the mRNA expression of genes specific to LD proteins like perilipin, adipophilin, TIP47, and FSP27 by 3.5-, 7.8-, 4.1- and 6.4-folds, respectively, was observed during HFFD feeding compared to CON. However, treatment groups showed reduced expression of LD proteins, compared to HFFD groups. The expression of perilipin, adipophilin, TIP47, and FSP27 was 1.4-, 3.5-, 1.9- and 2.5-folds in HFFD + GSP, and 2.5-, 5.5-, 3.5- and 4.9-folds in HFFD + MET with respect to CON. In GSP + MET group, the expression of perilipin, adipophilin, TIP47, and FSP27 was 1.5-, 3.7-, 1.9- and 2.5-folds respectively, with respect to CON (Figure 7) (see Figures 7A, 7B, 7C and 7D in Supplementary Material available online at http://dx.doi.org/10.1155/2013/153897).

**Figure 8:**
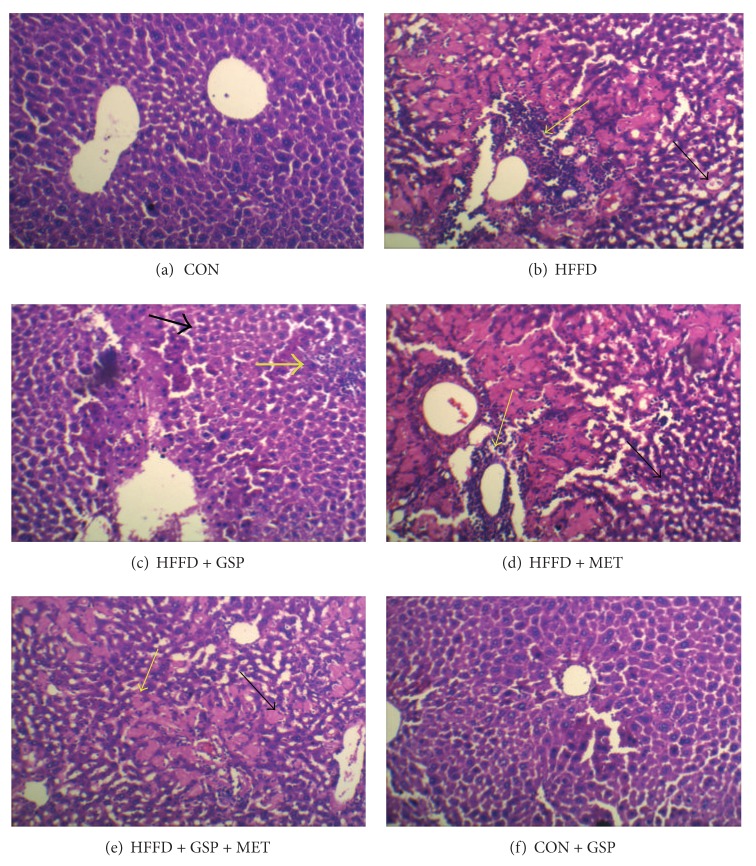
Histology of liver stained using hematoxylin and eosin. Section from control liver (a) and CON + GSP (f) shows hepatocytes within normal architecture arranged in the lobules around the central vein. Liver of HFFD-fed rats (b) shows severe micro- and macrovesicular changes and dense perivascular inflammatory infiltrate. HFFD + GSP show minimal inflammatory infiltrate with mild steatosis (c). Liver section from HFFD + MET (d) shows moderate amount of inflammatory infiltrate with microvesicular degeneration of hepatocytes. Section from the liver of HFFD + GSP + MET (e) shows hepatocytes with marked reduction of inflammation and steatosis. (Black arrows indicate steatosis and the yellow arrows indicate inflammation.)

The extent of inflammation and steatosis was examined histologically in liver sections using hematoxylin and eosin staining. The representative photographs show (Figure 8) severe micro- and macrovesicular fatty changes with dense perivascular inflammatory infiltration in tissues from HFFD-fed rats (Figure 8(b)). Upon GSP administration to HFFD-fed rats, the inflammatory infiltration is reduced to a minimum with mild steatosis (Figure 8(c)). HFFD + MET group of rats show moderate amount of inflammatory infiltration with microvesicular degeneration of hepatocytes (Figure 8(d)). HFFD + GSP + MET group of rats show hepatocytes with marked reduction of inflammation and steatosis (Figure 8(e)). Liver architecture of GSP-treated control rats (Figure 8(f)) appears normal as that of control rat liver (Figure 8(a)).

**Figure 9:**
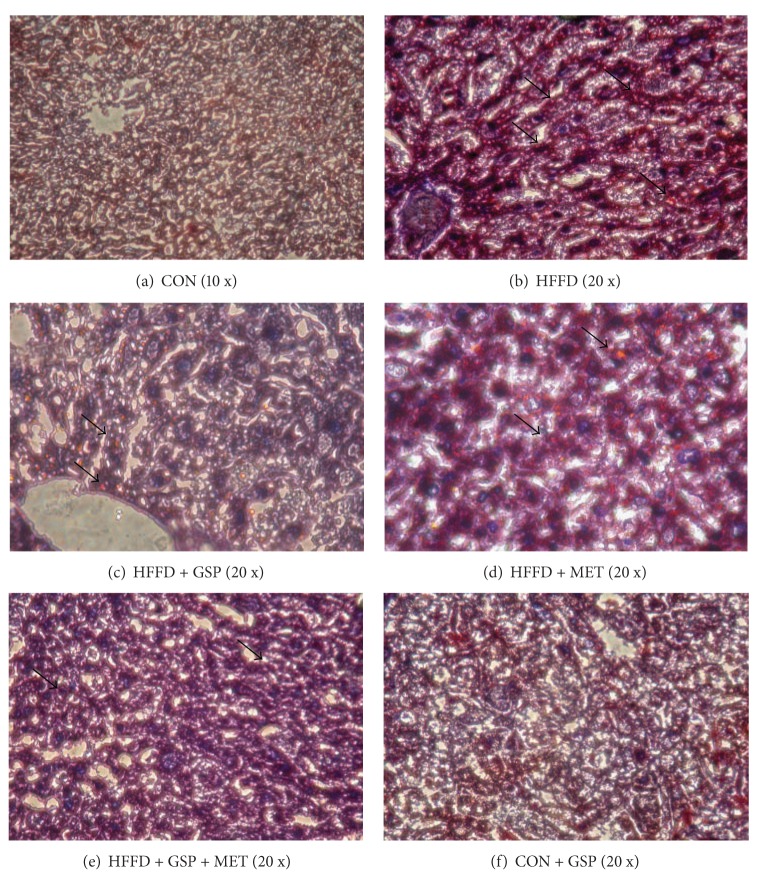
Histology of liver stained using Oil Red O. Sections from liver tissue of CON (a) and CON + GSP (f) show liver sections with normal architecture. HFFD-fed rats show severe steatosis and ballooning degeneration of hepatocytes (b). GSP administration to HFFD-fed rats show, minimal amount of steatosis (c), whereas MET administration to HFFD-fed rats shows moderate steatosis (d). HFFD + GSP + MET show hepatocytes with occasional areas of steatosis (e). (Black arrows indicate Oil Red stain accumulation.)

Figure 9 shows accumulation of LDs in liver sections examined by Oil Red O staining. Sections from liver tissue of HFFD-fed rats show severe steatosis and ballooning degeneration of hepatocytes (Figure 9(b)). HFFD + MET showed moderate steatosis (Figure 9(d)), whereas HFFD + GSP showed minimal amount of steatosis (Figure 9(c)). HFFD + GSP + MET showed hepatocytes with occasional areas of steatosis (Figure 9(e)). CON (Figure 9(a)) and CON + GSP (Figure 9(f)) show liver sections with normal lipid distribution.

## 4. Discussion

The present study examined whether the supplementation of GSP, MET, or both could exert protective effects against HFFD-induced obesity and NAFLD in Wistar rats. The expression profiles of genes related to lipogenesis and fatty acid oxidation in liver were examined. The combination of GSP with MET decreased hepatic lipid accumulation significantly. Between the two, administration of GSP showed more favorable effect on hepatic steatosis in HFFD-induced obese rats as compared to MET. However, combination treatment did not have significant additive benefit over treatment with GSP alone. 

Altered plasma lipid profile and lipoprotein abnormalities like decreased HDL-C and increased LDL-C, and VLDL-C were observed in HFFD-fed rats. Abnormalities in circulating plasma lipoproteins account for the increased depots of TG in liver. Accumulation of TG, a chief matrix of the lipid droplets in hepatocytes, is accelerated under conditions such as insulin resistance or hypertriglyceridemia, if left without appropriate treatment.

The potential relationship between the LD proteins and NAFLD has been explored experimentally. Accumulation of intracellular LDs in nonadipose tissues is recognized as a strong prognostic factor for the development of insulin resistance in obesity. A recent study has shown that reducing ADRP and TIP47 in the liver via antisense oligonucleotide treatment attenuated steatosis and improved insulin sensitivity and glucose metabolism in C57BL/6J mice fed with high-fat diet [24]. Knockdown of FSP27 in the ob/ob mouse liver partially improved the fatty liver [25] suggesting that FSP27 plays a vital role in the development of liver steatosis. 

Given the importance of PAT family proteins in NAFLD, we investigated their status in HFFD-fed rats, and in the treatment groups. In HFFD rats we observed that GSP reduces the levels of the LD-specific proteins. Reduction in PAT proteins and FSP27 suggests lipolysis of the stored TG. Also, considering the reduced TG content in liver and plasma and liver histology using oil red O, it is clear that GSP has the ability to reduce TG storage and LD formation. MET reduces the LDs formation but is less efficient than GSP. Histological examination of liver using hematoxylin and eosin and Oil Red O staining showed increased accumulation of inflammatory cells and LDs, with macrovesicular fatty changes and hepatocyte ballooning in liver of HFFD group of rats. GSP administration to NAFLD rats reduced steatosis to microvesicular fatty changes and inflammatory cell infiltration whereas MET showed reduced inflammation. However, in MET + GSP group, the expected combined positive effects were not observed, the reason for which is yet to be explored.

SREBP-1c controls hepatic *de novo* lipogenesis (DNL) primarily by regulation of expression of genes involved in DNL and lipid homeostasis [26]. Therefore, this transcription factor is a favored candidate in experiments that investigate the role of hypolipidemic agents in the prevention of fatty liver disease. Similarly, HMG CoA reductase is an enzyme of much importance in lipogenesis since it catalyzes the rate-limiting step in cholesterol biosynthesis. Increase in this enzyme indicates increased production of cholesterol. The observed increase in mRNA expression of SREBP-1c and HMG CoA reductase in HFFD-fed rat liver was reduced after GSP treatment. 

PPAR-*α* mediates *β*-oxidation of fatty acids, and agonists of PPAR-*α* possess the property of reducing lipid dystrophy and obesity [27]. PPAR-*α* activates some of the key enzymes of *β*-oxidation and *ω*-oxidation of fatty acids in liver [28]. HFFD-fed rats displayed reduced expression of PPAR-*α* in liver. Multitude of studies have postulated that, during diet-induced obesity, fatty acid oxidation is left incomplete accompanied by mitochondrial lipid overload and dysfunction [29, 30]. GSP treatment reduced the levels of FFA in plasma and liver and also improved the expression of PPAR-*α*. The lipid regulatory mechanism of GSP on lipid metabolism is observed to be at the genetic level. Additionally, GSP may improve cellular fatty acid uptake and mitochondrial function which needs to be studied in future. 

In conclusion, we have shown that GSP protects against hepatic steatosis in obesity by suppressing lipogenesis and promoting *β*-oxidation in liver. GSP reduces LD formation by reducing hepatic TG content, regulates lipid biosynthesis, and promotes *β*-oxidation through its effects on SREBP-1c, LD proteins, and PPAR-*α* mRNA expression. Since NAFLD and insulin resistance are closely associated, we linked and studied the action of GSP along with MET, an insulin sensitizer, on lipid abnormalities. Although MET showed significant restoration of lipid levels, comparative results point out that GSP is more effective. Histopathological examination provides good evidence for the lipid-lowering property of GSP whereas MET does not show a strong action against lipid abnormalities. Combination of MET with GSP showed effective improvement in insulin sensitivity and better reduction in hyperlipidemia compared to MET alone. The effects of MET and GSP on the parameter studied are not additive during combination treatment, the reason for which is unclear at this stage. Combination of drugs with different mechanisms of action has always been encouraged by clinicians in order to obtain the best results. Further studies are warranted to attest their putative positive effects of GSP and MET on other components of NAFLD and to see if NAFLD can be dealt using the combination of these two drugs. 

## Supplementary Material

Supplementary Figure 5: Effect of GSP and MET on mRNA expression of lipogenesis markers in liver of experimental animals. mRNA of SREBP-1c (a) and mRNA of HMG CoA reductase (b). Experiments were performed in triplicates, and the data expressed are means ± S.D. of 3 rats from each group. The obtained Ct values of the test genes were normalized with GAPDH and expressed in bars as fold change. Statistical significance between the groups, denoted by different alphabets, was determined by one-way ANOVA of significance set at *P < 0.05*.
Supplementary Figure 6: Effect of GSP and MET on mRNA expression of fatty acid oxidation marker PPAR-*α* in liver of experimental animals. Experiments were performed in triplicates, and the data expressed are means ± S.D. of 3 rats from each group. The obtained Ct values of the test gene were normalized with GAPDH and expressed in bars as fold change. Statistical significance between the groups, denoted by different alphabets, was determined by one-way ANOVA of significance set at *P < 0.05*.
Supplementary Figure 7: Effect of GSP and MET on mRNA expression of LD proteins perilipin (a), adipophilin (b), TIP47 (c), and FSP27 (d) in liver of experimental animals. Experiments were performed in triplicates and the data expressed are means ± S.D. of 3 rats from each group. The obtained Ct values of the test genes were normalized with GAPDH and expressed in bars as fold change. Statistical significance between the groups, denoted by different alphabets, was determined by one-way ANOVA of significance set at *P < 0.05*.

## Figures and Tables

**Table 1:** List of primers used in the study.

Genes	Forward →3′-5′	Reverse →3′-5′
SREBP-1c	gcctatttgatgccccctat	cccagagaagcaggagaaga
HMG CoA reductase	tgcttggtttctggctcttt	ttaacccattggaggtgagc
PPAR-*α*	cgttttggaagaatgccaag	gccagagatttgaggtctgc
Perilipin	gagcgaattccaagacatcg	tgtctcggttttgtcatcca
Adipophilin	tccgcaatgttacctccttc	aagggacctaccagccagtt
TIP47	atgtgttcccccaaactgag	tgtaggcagcactcaccatc
FSP27	actgcagtggtgacccaac	atgatgcctttgcgaacct
GAPDH	aaggggaacccttgatatgg	cggagatgatgacccttttg
