# Supplementary material for: Grape Seed Proanthocyanidin Rescues Rats from Steatosis: A Comparative and Combination Study with Metformin
Source: J Lipids. 2013 Nov 6;2013:153897. doi: 10.1155/2013/153897 (PMC3836386; doi:10.1155/2013/153897)
Supplement: Supplementary file 1 — Supplementary Figure 5: Effect of GSP and MET on mRNA expression of lipogenesis markers in liver of experimental animals. mRNA of SREBP-1c (a) and mRNA of HMG CoA reductase (b). Experiments were performed in triplicates, and the data expressed are means ± S.D. of 3 rats from each group. The obtained Ct values of the test genes were normalized with GAPDH and expressed in bars as fold change. Statistical significance between the groups, denoted by different alphabets, was determined by one-way ANOVA of significance set at P < 0.05. Supplementary Figure 6: Effect of GSP and MET on mRNA expression of fatty acid oxidation marker PPAR-α in liver of experimental animals. Experiments were performed in triplicates, and the data expressed are means ± S.D. of 3 rats from each group. The obtained Ct values of the test gene were normalized with GAPDH and expressed in bars as fold change. Statistical significance between the groups, denoted by different alphabets, was determined by one-way ANOVA of significance set at P < 0.05. Supplementary Figure 7: Effect of GSP and MET on mRNA expression of LD proteins perilipin (a), adipophilin (b), TIP47 (c), and FSP27 (d) in liver of experimental animals. Experiments were performed in triplicates and the data expressed are means ± S.D. of 3 rats from each group. The obtained Ct values of the test genes were normalized with GAPDH and expressed in bars as fold change. Statistical significance between the groups, denoted by different alphabets, was determined by one-way ANOVA of significance set at P < 0.05. [file 153897.f1.pdf]

Supplementary files

For Figure 5A

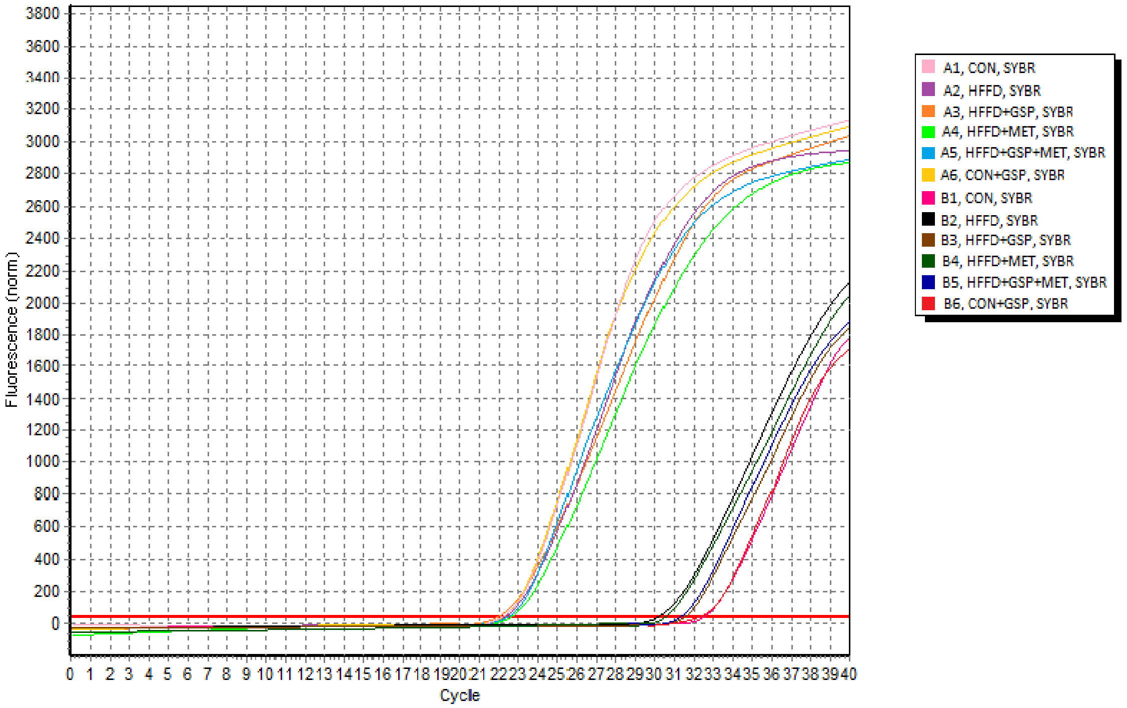

Threshold: 50 (Adjusted manually)

Baseline settings: automatic, Drift correction OFF

For Figure 5B

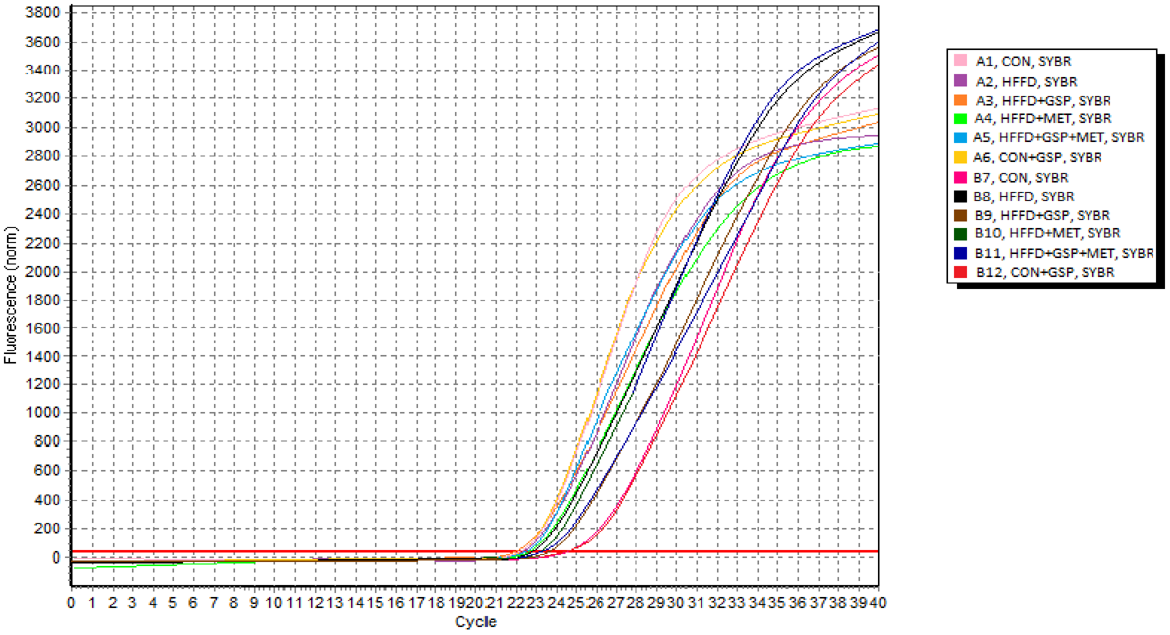

Threshold: 50 (Adjusted manually)

Baseline settings: automatic, Drift correction OFF

For Figure 6

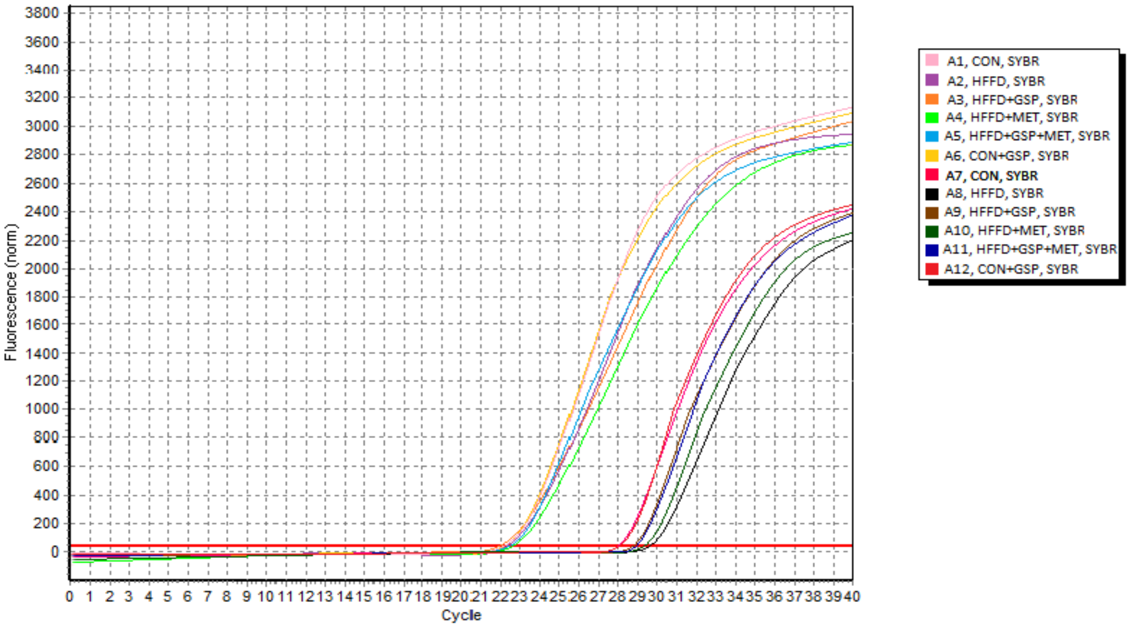

Threshold: 50 (Adjusted manually)
Baseline settings: automatic, Drift correction OFF

For Figure 7A

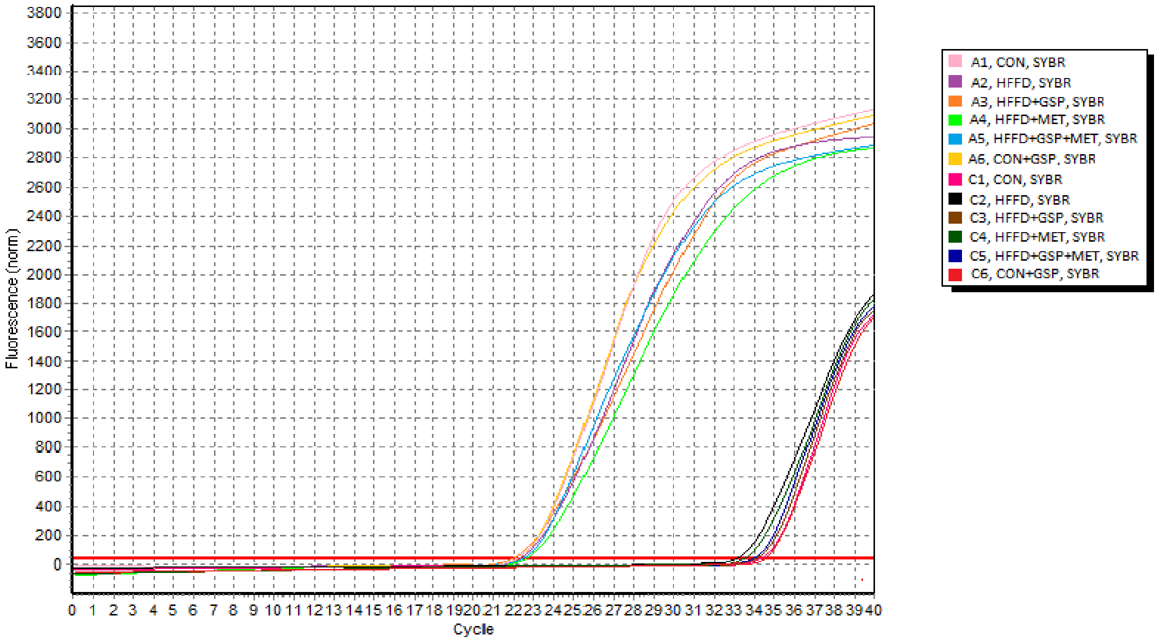

Threshold: 50 (Adjusted manually)
Baseline settings: automatic, Drift correction OFF

For Figure 7B

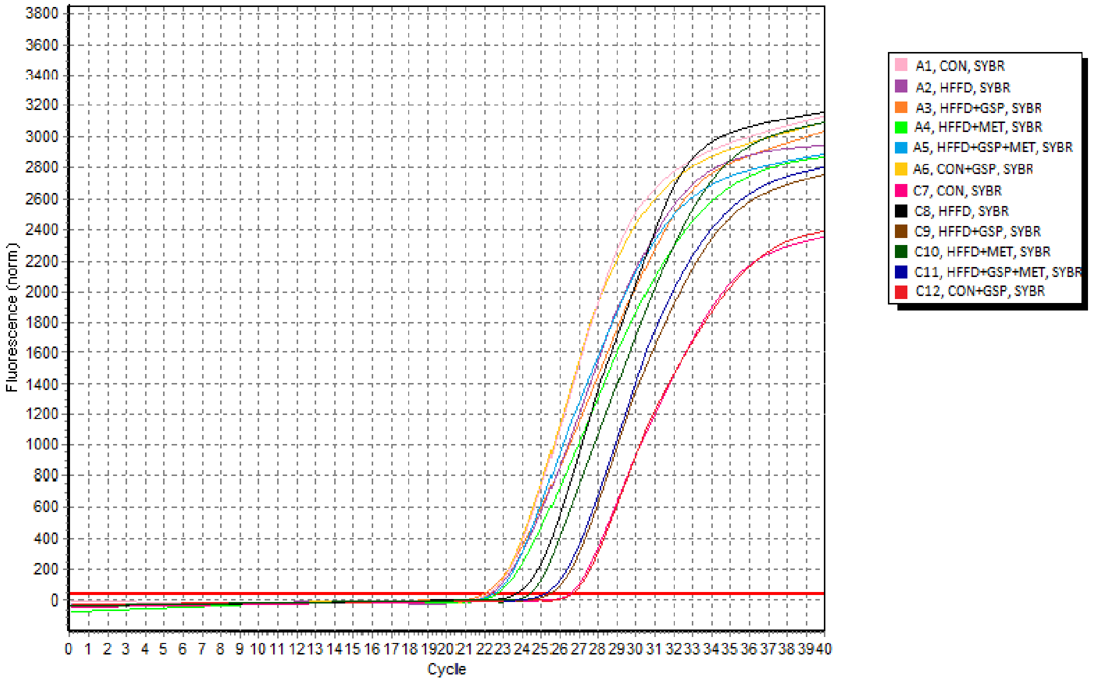

Threshold: 50 (Adjusted manually)
Baseline settings: automatic, Drift correction OFF

For Figure 7C

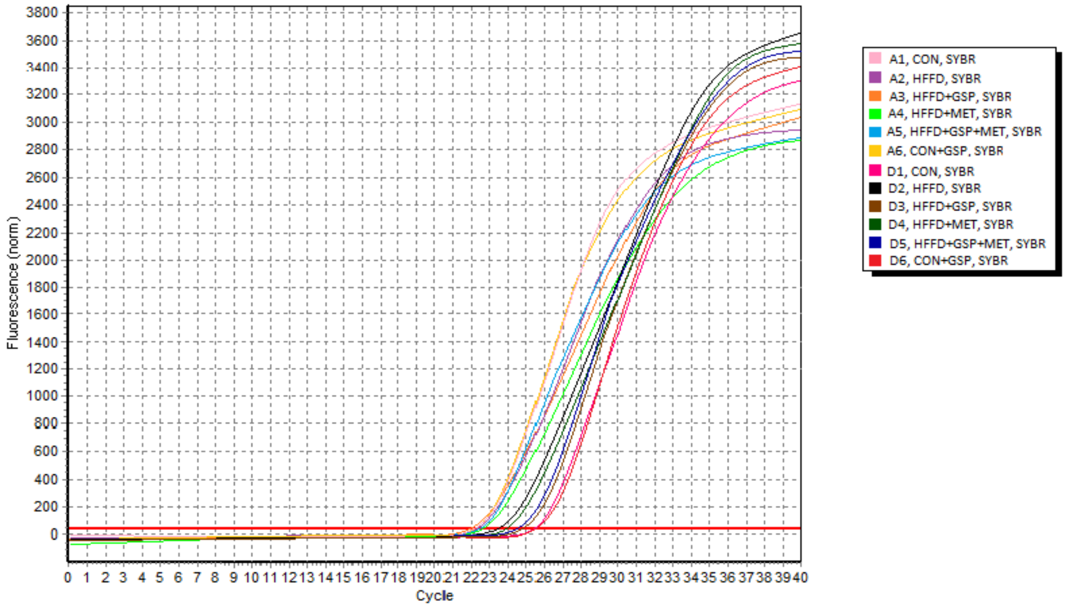

Threshold: 50 (Adjusted manually)
Baseline settings: automatic, Drift correction OFF

For Figure 7D

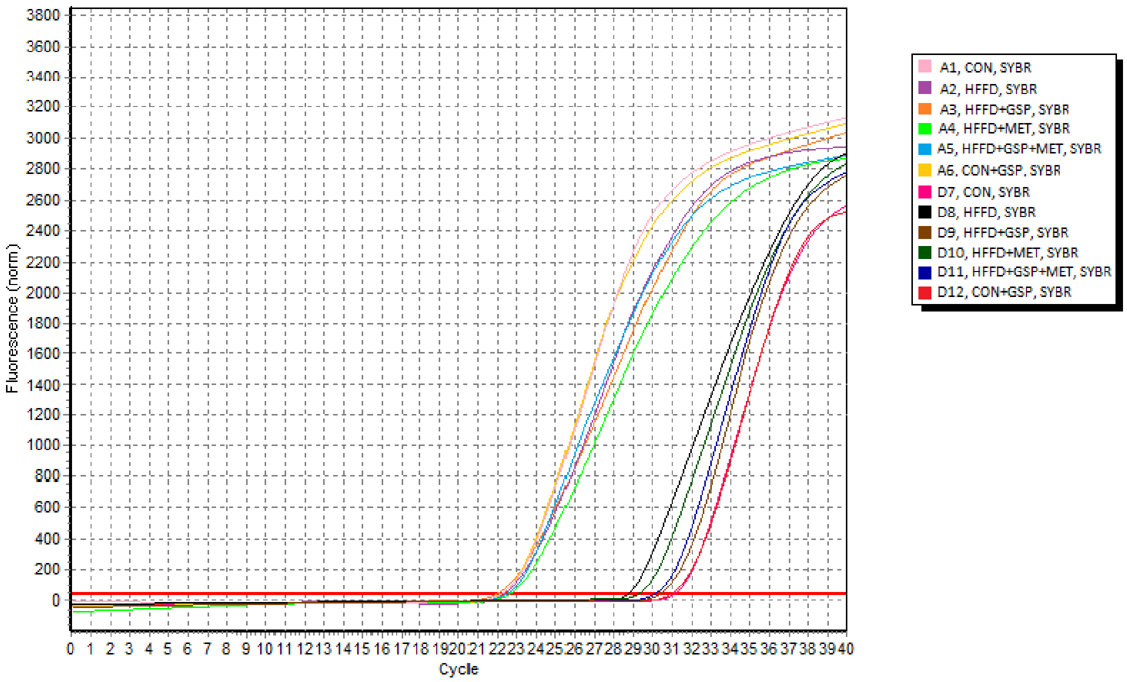

Threshold: 50 (Adjusted manually)
Baseline settings: automatic, Drift correction OFF
